# Abortion and Zika Virus Congenital Infection

**DOI:** 10.1055/s-0038-1668531

**Published:** 2018-08

**Authors:** Pathum Sookaromdee, Viroj Wiwanitkit

**Affiliations:** 1 Medical Center, TWS Medical Center, Bangkok, Thailand; 2 Department of Community Medicine, Faculty of Medicine, DY Patil University, Pune, India

Dear Editor,

We read the publication on “Abortion in Cases of Zika Virus Congenital Infection.”^1^ Mota et al^1^ noted that “*it is necessary to have available tests that could diagnose, in the first trimester of pregnancy, that the fetus has been affected by the virus, and that it may have important limitations, in order to subsidize the qualified discussion about abortion in these cases.*” In fact, there is still no evidence that the Zika virus infection in pregnant women is the cause of abortion, and there is still no recommendation for therapeutic abortion in infected pregnant women. The important issue is the relationship between infection during pregnancy and the induction of an abnormal infant. In many regions, especially in tropical Asian countries,^2^ ^3^ microcephalic infants are not the common finding in cases with a history of infection during pregnancy. Hence, the recommendation for abortion is not set. In the area where asymptomatic infection is very common, the early diagnosis might be useful for some purposes, such as epidemiological monitoring, but it should not be the presumptive data for decision on abortion for the pregnant woman infected with Zika virus.
